# Prevalence, socio-demographic features and risk factors of Hepatitis B virus infection among pregnant women in Southwestern Nigeria

**DOI:** 10.11604/pamj.2015.20.406.6206

**Published:** 2015-04-24

**Authors:** Chinenye Gloria Anaedobe, Adeola Fowotade, Chukwuma Ewean Omoruyi, Rasheed Ajani Bakare

**Affiliations:** 1 Institute of Human Virology Nigeria, FCT, Abuja, Nigeria; 2 Department of Medical Microbiology and Parasitology, University College Hospital, Ibadan, Oyo State, Nigeria; 3 Institute of Child Health, College of Medicine, University of Ibadan, Oyo State, Nigeria; 4 Department of Medical Microbiology and Parasitology, University of Ibadan, Oyo State, Nigeria

**Keywords:** Hepatitis B virus, Hepatocellular cancer, prevalence, risk factors, pregnancy, vertical transmission, serology, molecular, Ibadan

## Abstract

**Introduction:**

Hepatitis B virus is responsible for 50%-80% of Hepatocellular carcinoma cases worldwide. In Nigeria, vertical transmission remains a major route of Hepatitis B virus infection. Primary (vaccines and post-exposure prophylaxis) and secondary prevention of HBV transmission by appropriate sexual and sanitary practices are not yet optimal in the country yet measures for early detection (serological, molecular) and treatment of infected pregnant women is not a practice. This study aimed at identifying the prevalence and risk factors for Hepatitis B virus infection among pregnant women in Ibadan, Southwestern Nigeria.

**Methods:**

A cross-sectional study was done at the Ante-natal clinic of the University College Hospital Ibadan. One hundred and eighty pregnant women were recruited from March to August 2013, and tested for Hepatitis B surface antigen (BIORAD FRANCE) using third generation ELISA, as well as HIV-1 and 2 using Uni-Gold Recombigen and ALERE determine (a rapid immunoassay designed to detect antibodies to HIV 1 and/or 2). Positive HBsAg samples were tested for Hepatitis B envelope antigen, antibody and Hepatitis B core antibody (DIAPRO Italy) while serum HBV DNA was detected using PCR. Data were obtained using questionnaires to establish and analysis was performed using SPSS version 20.

**Results:**

The seroprevalence of HBsAg was 8.3% out of which 26.7% were positive for HBeAg, 53.3% had HBeAb, 20% had neither HBeAg nor HBeAb, 100% had total HBcAb and 86.7% had HBV DNA in their serum. The mean age was 32.1years, the highest HBV infection rate occurred in 25-29 year age group. Multiple sexual partners (OR- 3.987, P- value=0.026) and early age at sexual debut (OR 11.996, P- value=0.022) were independent risk factors for HBV infection.

**Conclusion:**

Hepatitis B virus infection is of high endemicity in Nigeria thus early detection, treatment of infected pregnant women, immunoprophylaxis for exposed newborns and surveillance for those with chronic infection is essential. Health education programs on prevention and control measures must be instituted.

## Introduction

Hepatitis B virus is a major cause of Chronic Hepatitis, cirrhosis and Hepatocellular cancer (HCC). About 2 billion people worldwide have been infected with HBV, an estimated 360 million remain chronically infected of whom almost one million people die annually of HBV-related liver disease [1, 2]. Of the estimated 360 million chronically infected individuals, about 50% acquired their infections either perinatally or in early childhood [2]. This is seen in sub-Saharan Africa and other HBV endemic regions, where high rates of HBeAg-positive infections in women of child-bearing age are found [2], and is considered a major contributing factor to the high prevalence of HBV infection in these regions [3]. Despite advent of anti-viral therapies that can suppress HBV and delay progression of liver disease, most people with chronic HBV infection reside in developing countries where these drugs are neither affordable nor accessible[4]. The incidence of HBV-related HCC cases is projected to increase for at least two decades due to the high prevalence of chronic HBV infection throughout the world [4]. Regional studies in Nigeria have shown varied sero-prevalence rates, ranging from 4.7% to 15.8% [5, 6]. Till date there has been no national study on the sero-prevalence of HBV in the Nigerian population as well as the pregnant women. Most regional studies done were solely on sero-prevalence of HbsAg, a few studies analyzed other serological parameters of HBV infection but no molecular study on HBV infection among pregnant women has been reported so far. In developed countries, the increased awareness, identification of mothers who are Hepatitis B surface antigen (HBsAg) and HBV DNA positive as well as adequate prophylaxis among exposed newborns was found to reduce the overall prevalence of HBV infection [7]. This must be emulated in Nigeria to ensure Prevention of perinatal transmission and hence decrease the burden of chronic HBV infection in Nigeria. In Nigeria, primary (vaccines and post-exposure prophylaxis) and secondary prevention of HBV transmission (appropriate sexual and sanitary practices) are not yet optimal in the country yet early detection (serological, molecular) and treatment of infected pregnant women is not a practice. Molecular test is necessary for accurate identification of pregnant women whom though asymptomatic have HBV infection and are likely to transmit the virus to their newborn and household. With occult hepatitis on the rise, molecular diagnosis of HBV infection becomes a necessity especially in an endemic region like Nigeria [8]. This study was set out to determine prevalence of Hepatitis B virus infection among pregnant women using both serological and molecular methods, as well as risk factors for acquisition of the disease. The findings are expected to preempt the review of Obstetrics and immunization policies in Nigeria with respect to routine screening and confirmation tests for HBV infection among Nigerian pregnant women attending antenatal clinics all over the country, as well as vaccination among women of reproductive age prior to conception.

## Methods

**Study design:** This was a cross-sectional study in which consenting pregnant women, attending Ante natal clinic at the University College Hospital in southwestern Nigeria were recruited between March2013 and August 2013 and their blood samples taken for analysis. Semi structured questionnaires which had been pretested was used to obtain socio-demographic characteristics and risk factors for HBV infections.

**Study population:** They were pregnant women attending antenatal clinic in the University College Hospital, Oyo State southwestern Nigeria and hailed from different parts of the country but largely comprised of the indigenes of Southern Nigeria. Sample size was calculated to give a 95% confidence level, a margin of error of +/-5%, using a previous survey of HBV seroprevalence found among pregnant women attending antenatal clinic in a central hospital in Warri, Delta State, southern Nigeria which was taken as 12%. A total of a hundred and eighty consenting pregnant women with ages ranging between 22 and 44 years were recruited. Women with prior vaccination, those who were unwilling to give consent for the blood test, and women diagnosed with Hepatitis B virus infection were exempted from the study. A written informed consent was obtained after careful explanation, in a clear language, of the concept of the study to each pregnant woman before their inclusion in the study. Ethical clearance was sought and obtained from the Joint Ethical committee of the University of Ibadan and University College Hospital Ibadan before the commencement of the study.

**Specimen collection and handling:** About 5 mls of venous blood was collected and transported to the laboratory. The blood was allowed to clot and sera separated by centrifugation at room temperature at 3000 rpm and stored in the freezer at -20°C. This was done on every visit to the Ante natal clinic.

**Laboratory investigations:** All samples were screened, using a sandwich third generation enzyme linked immunosorbent assay ELISA for HBsAg (BIORAD), as well as HIV-1 and 2 using Uni-Gold Recombigen and ALERE determine (a rapid immunoassay designed to detect antibodies to HIV 1 and/or 2). All samples found to be positive for HBsAg were further tested for HBeAg / HBeAb, total HBcAg (IgM and IgG) using ELISA kits (DIA-PRO manufactured by Diagnostic Bioprobes Milano Italy), and HBV DNA using a thermocycler to run the polymerase chain reaction and gel electrophoresis to identify the amplified gene of interest. All tests were carried out according to the manufacturer´s instructions as outlined in the package inserts.

**HBV detection by enzyme linked immunoassay:** A third generation enzyme linked immunoassay was used to identify presence of HBsAg (BIORAD FRANCE), HBeAg and HBe Antibody, HBcAg (DIAPRO ITALY). It is a solid-phase simultaneous sandwichimmunoassay, which employs specific monoclonal antibodies and polyclonal antibodies. Protocol for the measurement was done according to the manufacturer's instruction and reading was done at O.D. of 450 nm with an EIA plate reader. The tests ran were validated and results were interpreted according to the manufacturer's instruction.

**HBV DNA Isolation:** The E.Z.N.A blood DNA Mini Kit was used for DNA extraction in accordance with manufacturer's instructions. About 200 μl of serum was transferred into a sterile microcentrifuge tube and brought up to 250 μL with 10mM Tris-HCl. About 250 μl of BL buffer and 25 μl of proteinase K were added to the mixture and vortexed. Incubation was done at 65°C for 10 minutes to inactivate the proteinase, thereafter 260 μl of 100% ethanol was added and vortexed. The ethanol-buffer-specimen mixture was then placed into a HiBind^®^ DNA Mini column and centrifuged at 13,000 rpm for 1 minute and the flow through was discarded with the collection tube. About 500μl of HBC wash buffer was added, sample centrifuged at 13,000 rpm for 1 minute and filtrate discarded, after which 700μl of DNA wash buffer was added and centrifuged at 13,000 rpm for 1 minute, this was done twice and the flow through discarded. About 100 μl of preheated elution buffer was added, and centrifuged at 13,000 rpm for 1 minute, this was done twice to elute the DNA.

**HBV DNA amplification and detection:** Primer sequences used for HBV were Hep B gen 1 5’ TGC GGG GTT TTA TCA TCT TCC T-3’, Hep B gen 2 5’-GTT TAA ATG TAT ACC CAA AGA C-5’ and Hep B gen 3 5’-CAG CGG CAT AAA GGG ACT CAA G-5’. PCR round one set up comprised; 3μl DNA extract, 1.5 μl of primers Hep gen 1 and 2, 15.5 μl of PCR water were added to the BioneerAccu power Taq PCR premix (MgCl2, dNTPs, Taq polymerase, Taq buffer, reaction buffer) all in a tube. While the PCR round 2 was 2μl DNA extract, 36 μl of PCR water, 2μl of primers Hep gen 2 and 3. All tubes were sealed and briefly centrifuged before amplification in PCR machine (Robocycler gradient 40). Amplification was done with the following protocol; pre denaturation at 95°C for 5minutes, then 30 cycles of denaturation at 95°C for 30secs, annealing at 50°C for 30secs, extension at 72°C for 40secs and final extension at 72°C for 5 minutes. Each amplification run contained one negative and one positive control. After amplification, electrophoretic separation of PCR products was performed on 1.5% agarose gel stained with ethidium bromide, and visualized by UV illumination.

**Data analysis:** Data collected were subjected to descriptive and inferential statistical analysis using SPSS version 20. (SPSS Inc.Illinios, USA). The Mean, standard deviation and test of comparison using student´s t-test was derived for continuous variables, while categorical variables were summarized as proportions, and further analyzed using Chi square and Fisher´s exact test to assess association between the variables. Test of association using logistic regression was done to describe the relationship between the predictor variables (risk factors for maternal infection found to be statistically significant) and the outcome variable (HBsAg). P values ≤ 0.05 was considered significant.

## Results

**Socio-demographic characteristics of the participants:** During the study period, 190 consenting pregnant women were selected using a systematic random sampling method. Of these, 10 were excluded; among the reasons for exclusion were prior HBV vaccination and known HBsAg sero-positive status. The mean age of the one hundred and eighty participants finally enrolled was 32 (SD 4.8) years. The youngest was 22 years and the oldest was 44 years and more than half (55.6%) aged between 29 and 35 years. Almost all the women 172 (95.6%) were married and 172 (95.6%) were in a monogamous relationship. All participants had some level of education, with more than half of them 119 (66.1%) having tertiary-level education and about 77 (42.8%) of them were employed in the formal sector while 33 (18.3%) were unemployed. Multiparous respondents constitute the majority with 127 (70.6%) of the participants, more than half of them 111 (61.7%) were in their second trimester. The socio-demographic characteristics are as illustrated in Table 1.

**Table 1:** Association between socio-demographic factors and sero-prevalence of HBsAg among the pregnant women attending ANC at the University College Hospital Ibadan N=180

Variable	HBsAg positive (N%) N=15	HBsAg negative (N%) N=165	X2	P value
**Age group (years)**				
22-28	2(13.33%)	38(23.03%)	2.095	0.351
29-35	11(73.33%)	89(53.94%)		
≥ 35	2(13.33%)	38(23.03%)		
Type of Family				
Monogamous	15(100%)	157(95.15%)	0.761	0.383
Polygamous	0(0%)	8 (4.85%)		
**Level of Education**				
Primary	0(0%)	5 (3.03%)	6.532	0.038*
Secondary	9(60%)	47(28.48%)		
Tertiary	6(40%)	113(68.48%)		
**Marital Status**				
Married	15(100%)	157(95.15%)	0.761	0.383
Single	0(0%)	8 (4.85%)		
Religion				
Christian	11(73.33%)	126(76.36%)	0.069	0.502
Islam	4(26.67%)	39(23.64%)		
**Occupation**				
Employed (Govt/Private)	6(40%)	71(43.03%)	0.060	0.971
Self Employed	6(40%)	64(38.79%)		
Unemployed	3(20%)	30(18.18%)		
Gestational age				
1st trimester	4(26.67%)	27(16.36%)	1.684	0.431
2nd trimester	7(46.67%)	104(63.03%)		
3rd trimester	4(26.67%)	34(20.61%)		
**Parity**				
Primiparous	4(26.67%)	49(29.70%)	0.061	0.533
Multiparous	11(73.33%)	116(70.30%)		

* Significant at 5% level of significance

**Overall HBV prevalence:** HBsAg was detected in fifteen (8.3%) of the participants. Of the HBsAg sero-positive women, four (26.7%) were positive for HBeAg; eight (53.3%) were positive for HBeAb, three (20%) were negative for both HBeAg and HBeAb, while all fifteen (100%) had total HBcAb (both IgM and IgG). However, thirteen (86.7%) of those found to be sero-positive also had HBV DNA in their serum. The overall HBV prevalence is illustrated in Table 2. The highest HBV infection rate occurred in the 25-29 year age group and majority of the women presented in 2nd and 3rd trimester for first antenatal care.

**Table 2:** HBV prevalence among the pregnant women attending ANC at the University College Hospital Ibadan

Variable	Frequency (%)
HBsAg (n=180)	
Positive	15(8.3%)
Negative	165(91.7%)
HBV envelope (n=15)HBeAg positive	4(26.7%)
HBeAb positive	8(53.3%)
Both HBeAg and HBeAb negative	3(20%)
Total HBcAb (n=15)	
Positive	15(100%)
Negative	0(0%)
HBV DNA (n=15)	
Positive	13(86.7%)
Negative	2(13.3%)

**Seroprevalence and its risk factors among the participants:** A positive association was noted between participants’ level of education and HBsAg sero-positivity (X2=6.532, P-value=0.038). Employment status, religion, gestational age and parity, on the other hand, did not impact significantly on the likelihood of having Hepatitis B virus infection. Participants’ previous contact with known HBV infected persons (X2=3.905, P-value= 0.048), increasing number of current sexual partners (X2=6.204, P-value= 0.013) as well as life-time sexual partners (X2=5.077, P-value= 0.024) and early age at first sexual intercourse (X2= 9.298, P-value= 0.002) were significantly associated with HBV infection. None of the HBsAgsero-positive pregnant women volunteered any previous history of blood transfusion, a finding that was not statistically insignificant (X2=2.639, P-value= 0.096). There was also no significant difference in presence of HBsAg between the HIV-positive and HIV-negative women. Likewise, previous history of sexually transmitted infection, self-reported consistent use of condom (within the last six months), body tattooing, unsafe injection, body piercing with sharp objects as well as history of surgical procedure, showed no significant impact on the likelihood of having Hepatitis B virus infection among these women. This is further illustrated in Table 3. In addition, risk factors were assessed for their association with HBV infection using bivariate logistic regression. P value of less than or equal to 0.05 was used with level of education, previous contact with persons with HBV infection, lifetime sexual partners, current sexual partner and age at sexual debut being significantly associated. Including these variables in a stepwise multivariate model of logistic regression, women with multiple sexual partners were four times more likely to acquire HBV infection (OR- 3.987, P- value=0.026) while women with early sexual debut (less than 15years of age) were twelve times more at risk for HBV infection (OR 11.996, P- value=0.022).

**Table 3:** Association between some selected risk factors and Hepatitis B virus infection among pregnant women in University College Hospital Ibadan

Variable	HBsAg positive(%)N=15	HBsAg negative(%)N=165	X2	P value
**Sexual contact with known HBV positive persons**				
Yes	2(13.33%)	5 (3.03%)	3.905	0.048*
No	13(86.67%)	160(96.97%)		
**Current sex partners**				
One	10(66.67%)	147(89.09%)	6.204	0.013*
More than one	5(33.33%)	18(10.01%)		
**Lifetime sex partners**				
One	5(33.33%)	104(63.03%)	5.077	0.024*
More than one	10(66.67%)	61(36.97%)		
**Blood Transfusion**				
Yes	0(0%)	25 (15.15%)	2.639	0.096
No	15(100%)	140 (84.85%)		
**Age at First sexual exposure**				
<15years	2(13.33%)	2 (1.21%)	9.298	0.002*
>15years	13(86.67%)	163(98.79%)		
**Condom use**				
Yes	5(33.33%)	57(34.55%)	0.009	0.584
No	10(66.67%)	108(65.45%)		
**Past STI 1**				
Yes	3(20%)	29(17.58%)	0.055	0.520
No	12(80%)	136(82.42%)		
**Surgical procedure**				
Yes	4(26.67%)	50(30.30%)	0.087	0.514
No	11(73.33%)	115(69.70%)		
**HIV Status**				
Positive	4(26.67%)	33(20%)	0.374	0.371
Negative	11(73.33%)	132(80%)		

* Significant at 5% level of significance ^1^ Sexually Transmitted infection

## Discussion

A hospital-based cross sectional study was carried out at the ante-natal clinic of the University College Hospital Ibadan in southwestern Nigeria. One hundred and eighty pregnant women were recruited, their serum analyzed for Hepatitis B virus, while socio demographic characteristics and risk factors for HBV infection was assessed with pretested questionnaires. We found an HBsAg seoprevalence rate of 8.3% among the pregnant women tested, indicating that HBV is highly endemic in Nigeria. This is similar to the 8.3% prevalence each, found among pregnant women in South East [6], and North East Nigeria [9], as well as 7.9% in North Central Nigeria [10]. This may be due to the similarity in the socio-demographic characteristics, as the four studies were hospital based in an urban setting and similar laboratory methods for the analyses. Lower prevalence rates ranging from 2.2%-6.7% were observed in Southern region of Nigeria [11–13], while other parts of Nigeria showed higher prevalence ranging from 9.3-15.8% [14–16]. The prevalence rate depicts a trend that follows a low prevalence from the southern parts of the country increasing to its highest of 15.8% in the northern parts. Level of health education on prevention practices, early seeking of health-care assistance and effective utilization of these health-care facilities may play a role in this trend. Likewise risk factors such as early marriage and high risk sexual behaviour may contribute. In regional studies done among pregnant women in different African countries, lower prevalence rate of 3.9% and 6.3% were recorded in Tanzania [17], 3.7% in Ethiopia [18], 6.2% in Sierra Leone, 6.5% each in Congo and Zambia were reported [19], while higher prevalence rate of 25.3% was observed in Cameroon [20]. In other parts of the world, various prevalence rates were observed among their pregnant women and this agrees with WHO epidemiological survey report that Global prevalence of HBV infection varies highest in Africa, Asia and Western Pacific (>8%) and lowest in Western Europe, North America and Australia, with prevalence rate in sub-Saharan Africa ranging from 9% to 12% [9, 16–22]. The presence of HBeAg indicates active viral replication in hepatocytes with high risk for developing hepatocellular carcinoma [23]. In the study, women positive for HBeAg accounted for 26.7%. HBeAg transverses the placenta and induces an immune tolerance to the capsid viral antigen and elimination of infected hepatocytes in the infected newborn is therefore impossible without intervention [24]. All the HBsAg sero-positive pregnant women tested positive for total HBcAb hence were chronically infected and had HBV DNA identified in 86.7% of them, majority (73.33%) of whom presented in their second and third trimester. In the absence of early detection and treatment in these pregnant women, the risk of transmitting HBV to their newborns at birth is nearly 100% [24]. In our study, a high frequency of HBV infection was found among women older than 29 years. This may be due to increased risk of exposure to HBV with each pregnancy, the cumulative years of sexual exposure and risky sexual behaviour. This finding though in contrast to some other studies [6, 16], was consistent with a similar study done in the same region [25], all within Nigeria. Most of HBsAg-positive women were of low educational status (60%) and increasing level of education was noted to be inversely related with HBV infection, this finding was in agreement with similar studies in Nigeria [6, 10, 11, 16], and this indicates the positive influence of education and public awareness on the carrier rate of HBV infection.

About (73.34%) of the HBsAg positive women were multi-gravida, this finding concurs with the observation that pregnant women are considered at a higher risk of HBV infection due to increased exposure to risk factors (such as blood transfusion, intravenous drugs or surgical procedures) [26]. None of the participants with previous history of blood transfusion had HBV infection, this is similar to studies done in Southern Nigeria [5, 6, 25], and in contrast to some published studies in Northern Nigeria [10, 13, 27]. This reflects the strengthening of the national regulatory policy on universal screening of blood and blood product especially in Southern Nigeria and calls for further strengthening in the Northern part. In the index study women with multiple sexual partners were four times more likely to acquire hepatitis B virus infection than women with one sexual partner; this finding corroborates the high prevalence of HBV infection among commercial sex workers as reported in some studies [9]. It was also observed that women who had their sexual debut less than fifteen years of age were twelve times more at risk of HBV infection than those who were above fifteen years at their first sexual experience. Sexual contact with known HBV positive persons was also significantly associated with Hepatitis B virus infection among these women. Likewise, there was an increased prevalence of HBV infection among women in polygamous family setting, those whose husbands had other sexual partners and those whose husbands were unable to come home every day due to the nature of their job, thus reflecting the increased likelihood of their partners engaging in risky sexual behavior. This concurs with reports among pregnant women in Afghanistan [22], and buttresses findings in literature ascribing sexual activity as a major risk factor for HBV infection [9, 28]. HBV Co-infection with human immunodeficiency virus (HIV) increases the rate of transmission of viral hepatitis substantially. It also increases the risk for hepatotoxicity of HAART and likelihood of onset of an AIDS-defining illness [28, 29]. The HIV/HBV co-infection rate in this study was 26.67%, this is similar to a study done in Tanzania [17] and Nigeria [29], but quite high when compared with similar studies in Nigeria [6], and the literature [28, 30]. This study also highlighted that some of the respondents did not fall into any of the recognized high risk groups for HBV infection as was also reported by other authors [10, 14, 18]. This is not an uncommon finding as other studies have indicated that the sensitivity of Center for Disease Control and Prevention (CDC) risk factors for screening pregnant women for HBsAg ranges between 35 and 65% [10, 31]. This further buttress the fact that these women may have contracted the virus at birth, through household contacts or peer groups, or from unrecognized modes of transmission of HBV and this may herald even more widespread prevalence if not studied and curtailed. Our study was not without limitations. First, the results cannot be generalized to all pregnant women. As Pregnant women with prior history of HBV vaccination were not included in this study and known HBsAg positive women were also excluded from this study. However, only a minority of eligible patients were excluded for this reason. Second, the selection of participants for the study was limited to the first 180 consenting pregnant women who met the eligibility criteria and this may have introduced a bias. However, it is unlikely that bias was introduced owing the use of a randomized sampling technique in selection of participants.

## Conclusion

The result of this study brings to light the high prevalence and high infectivity rate of chronic Hepatitis B virus infection among women of child bearing in Nigeria. In our study, predictors of HBV infection include early age at first sexual intercourse and multiple sexual partners. Although level of education, HIV status and sexual contact with known HBV positive partners lost their significance on regression analysis; they were associated with increased prevalence of HBV infection in this study while blood transfusion, past surgical history, body tattooing, unsafe injection, body piercing with sharp objects and inconsistent use of condoms were not. The high prevalence of HBV in this study, underscores the importance of emulating global best practices towards curbing the spread of the infection. There is a dire need for routine screening of pregnant women during Antenatal care in Nigeria, to identify those with chronic infection who serve as a reservoir for person-to-person transmission of the virus as well as vertical transmission to their infants. The infected pregnant women may benefit from treatment, interventions to prevent transmission to their infants, as well as surveillance for hepatocellular carcinoma. If pregnant women are left undiagnosed and unmanaged, the future burden of the disease for healthcare resources and society will be substantial.
